# Long-term safety and efficacy of Canakinumab in cryopyrin-associated periodic syndrome (CAPS) patients: results from beta-confident registry

**DOI:** 10.1186/1546-0096-13-S1-P3

**Published:** 2015-09-28

**Authors:** J Kuemmerle-Deschner, H Hoffman, PN Hawkins, T van der Poll, UA Walker, A Speziale, HH Tilson

**Affiliations:** 1 University Hospital Tuebingen, Tuebingen, Germany; 2 University of California, La Jolla, CA, USA; 3 University College London Medical School, London, UK; 4 University of Amsterdam, Academic Medical Center, Amsterdam, Netherlands; 5 University Hospital, Basel, Switzerland; 6 Novartis Pharma AG, Basel, Switzerland; 7 University of North Carolina, Chapel Hill, USA

## Background

CAPS encompasses a spectrum of three phenotypes: familial cold auto-inflammatory syndrome (FCAS), Muckle-Wells syndrome (MWS), and chronic infantile neurologic cutaneous and articular syndrome/neonatal onset multisystem inflammatory disease (CINCA/NOMID)[^1^]. The β-Confident Registry, the largest CAPS cohort documented in a registry, enrolled the last patient in December 2014. Here, we report interim data for the complete cohort of enrolled patients.

## Objectives

To monitor the overall safety of canakinumab (CAN) focusing on SAEs including serious infections, vertigo, malignancies, and hypersensitivity reactions.

## Patients and methods

The registry protocol does not mandate any visits or procedures, but records all observed and reported AEs and SAEs or AEs potentially CAN-related. Cumulative safety data are reported as incidence rate per 100 patient-years (IR/100 pyr). Data is partial for 11 patients due to the cut-off date for the analysis and will be updated at a later date. Efficacy was measured using physician global assessments (PGA).

## Results

288 patients were enrolled with a mean±SD duration of 193±72 weeks. Of these, 21 (7.3%) patients discontinued CAN: 5 each due to AE, poor efficacy and patient preference; and 6 due to unknown reasons. The IR/100 pyr for overall AEs was 100.0. FCAS patients had the lowest AE IR/100 pyr (60.9) compared with MWS (IR/100 pyr 107.2) and NOMID (IR/100 pyr 120.3) patients. The most common types of AEs were infections and infestations (IR/100 pyr 36.7). Vertigo was reported by 19 patients (IR/100 pyr 3.7). 117 SAEs were reported by 62 patients (IR/100 pyr 15.0), with infection being the most common (IR/100 pyr 4.1). One death (metastatic rectal adenocarcinoma in 76 yr old MWS patient) was reported. Of 18 patients receiving pneumococcal vaccinations (PPV), 13 (72%) reported a local post-PPV injection site reaction, of which 5 were considered as serious. Based on PGA, nearly half the patients had no disease activity while most others had mild/moderate disease activity. Similarly, disease activity was mostly absent in *NLRP3* mutation negative CAPS patients (n=14) treated with CAN. There was no evidence of loss of effect with time. Further analyses of this cohort are ongoing.

## Conclusions

Canakinumab demonstrated a safety profile consistent with that observed in the clinical trial program and provided continued effectiveness in CAPS patients for up to 5 years. Canakinumab therapy was also effective in *NLRP3* mutation negative CAPS patients.
